# Microstructural and Electrochemical Influence of Zn in MgCaZn Biodegradable Alloys

**DOI:** 10.3390/ma16062487

**Published:** 2023-03-21

**Authors:** Bogdan Istrate, Corneliu Munteanu, Madălina-Simona Bălțatu, Ramona Cimpoeșu, Nicoleta Ioanid

**Affiliations:** 1 Mechanical Engineering, Mechatronics and Robotics Department, Mechanical Engineering Faculty, “Gheorghe Asachi” Technical University of Iasi, 700050 Iasi, Romania; 2 Technical Sciences Academy of Romania, 26 Dacia Blvd., 030167 Bucharest, Romania; 3 Department of Technologies and Equipments for Materials Processing, Faculty of Materials Science and Engineering, “Gheorghe Asachi” Technical University of Iasi, Boulevard D. Mangeron, No. 51, 700050 Iasi, Romania; 4 Materials Science Department, Faculty of Materials Science and Engineering, “Gheorghe Asachi” Technical University of Iasi, 700050 Iasi, Romania; 5 Faculty of Dental Medicine, “Grigore T. Popa” University of Medicine and Pharmacy, 700050 Iasi, Romania

**Keywords:** Mg–Ca–Zn alloys, microstructure, XRD analysis, corrosion resistance, EDS analysis

## Abstract

In recent years, biodegradable materials have included magnesium alloys with homogenous disintegration and a controllable degradation rate. Utilized in medical applications, biodegradable materials based on magnesium have been widely explored throughout the years. It is well-known that alloying Mg with biocompatible and non-toxic elements increases the biodegradability of surgical alloys. The purpose of this study was to examine the microstructure and the electrochemical response (corrosion resistance) of a new experimental Mg-based biodegradable alloy—Mg–0.5%Ca with additions of Zn as follows: 0.5, 1.5, and 3.0 wt.% in order to control the corrosion rate. Immersion tests were performed for different periods in a simulated body fluid electrolyte solution at 37 °C, and the mass loss was appreciated in order to calculate the corrosion rate (CR). The investigation led to the discovery of a dendritic Mg solid solution, a lamellar Mg_2_Ca compound, and a MgZn_2_ intermetallic phase. Scanning electron microscopy, optical microscopy, and energy dispersive spectroscopy were used for surface analysis after the immersion and electro-corrosion resistance tests. The metallic and ceramic compounds that detached themselves from the sample and passed into the solution were evaluated using the SEM-EDS system. All samples presented a generalized electro-corrosion with anodic and cathodic reactions of similar intensity. The corrosion rate was similar regardless of the percentage of zinc, with a smaller value for a higher than 3 wt.% Zn percentage based on the more protective zinc oxide that appeared on the surface.

## 1. Introduction

Currently, biomaterials constitute a special category of materials, indispensable for raising the quality of human life and extending its duration. Biomaterials, in general, are intended for implantation in a living organism to restore the form and function of a part of a tissue destroyed by disease or trauma. Despite the large number of metals and alloys able to be produced in industry, only a few are biocompatible and have long-term success as implant materials. Biomaterials are components in most commercially available orthopedic medical devices [1,2,3].

Magnesium has begun to be introduced more often in orthopedic applications. The major advantage offered by Mg alloys, which has brought Mg to the forefront of research attention, is its suitable mechanical properties compatible with human bone, its biocompatibility and biodegradability as well as a corrosion rate that can match that of bone tissue healing.

Magnesium is more commonly used than classic alloys (Ti or Co based alloys) to obtain bone implants because these materials present mechanical properties (density: 1.75 g/cm^3^, Young’s modulus: 41–45 MPa, ultimate strength: 95–185 MPa, yield strength: 65–95 MPa, elongation: 2–10%) similar to those of human bone tissues (density: 1.8–21 g/cm^3^, Young’s modulus: 15–25 MPa, ultimate strength: 110–130 MPa, yield strength: 104–121 MPa, Elongation: 0.7–3%). It also presents rates of corrosion between 1.63 and 12.5 mm/year and hydrogen evolution, which are drawbacks of utilizing magnesium for bone implants [4,5,6,7,8].

Biodegradable refers to a material that breaks down if placed in a biological environment. Obviously, this is a function of both the material and the environment chosen. A material that is biodegradable in some environmental conditions may not be biodegradable in others. A metal that degrades in vivo is a biodegradable material.

As a result of investigations in recent years, elements such as Ca, Zn, Zr, Nd, and Sn have proven to be very encouraging chemical components to be used in magnesium alloys because they gradually degrade in the human body like magnesium, without increasing the level of serum Mg^2+^ or damaging important internal organs such as the kidneys [9,10,11,12,13]. These specific components, especially calcium, aid in the prevention of corrosion in magnesium alloys.

Calcium is among the most significant and vital components of human bones in terms of chemical transmission with various cells. In addition, calcium is a cheap chemical element with a density equivalent to bone tissue (1.55 g/cm^3^) and has the capacity to create hydroxyapatite (HA) during corrosion in the human body, which accelerates bone healing. Calcium decreases the grain size of magnesium alloys, maintaining the grain size up to concentrations of 0.5% Ca and then reducing substantially as calcium concentrations increase [14]. Regarding binary Mg–Ca alloys, multiple studies [15,16] have revealed that the secondary phase Mg_2_Ca evolves at the grain boundary with an increase in calcium content for the investigated Mg-(0.5–20 wt.%) alloys; a phase that decreases the ductility even when the Mg_2_Ca phase is well-refined and dispersed. This secondary phase also decreases the corrosion resistance due to the formation of microgalvanic cells. Therefore, due to the negative effect on corrosion resistance, the amount of calcium in magnesium alloys should not exceed 1 wt.%.

The rate of corrosion of binary Mg–Ca alloys [17] has attracted much interest. The chemical range of binary Mg–Ca alloys is the topic of the first in-depth examination of these materials. Li et al. [18] and Bita et al. [19] noticed in their research on Mg–0.8Ca alloys that an increase in the CaMg_2_ phase improved the corrosion resistance. In addition, Antoniac et al. [20] examined the Mg–1Ca alloy as a possible material for minor bone fracture healing applications. Samples were implanted into the greater femoral trochanter of the Oryctolagus Cuniculus rabbit animal model. It has been shown that local tissue metabolism influences the corrosion process, and the Mg–1Ca alloy fulfills all biocompatibility criteria. During a fast X-ray examination of rabbits, it was found that the substance under investigation did not produce harmful by-products or gas bubbles after implantation in bone.

Zinc has the chemical ability to transform atoms or impurities in magnesium-based alloys such as iron (Fe), copper (Cu), and nickel (Ni) into harmful intermetallic compounds, thus reducing their beneficial effect on corrosion resistance. Studies have reported that zinc additions are associated with the reduction in grain size and the formation of secondary phases, thus influencing the mechanical and corrosion properties of magnesium alloys. Research has shown that the addition of 3 wt.% Zn to Mg–Zn–Mn alloys forms Mg–Zn secondary phases that precipitate in the alloy matrix, raising the mechanical strength through a dispersion hardening mechanism [7].

Nanda et al. [21] studied the corrosion behavior of a binary Mg–Zn alloy by testing five Mg–xZn alloys with x = 2, 4, 6, 8, and 10. A significant association was found between the Zn content and an increase in the corrosion potential value. The rapid disintegration process may be explained by the formation of the MgZn phase in the matrix of the alloy, which serves as a barrier against ion diffusion and lowers the alloy’s electrochemical contact with the electrolyte. Antoniac et al. claimed in their review study [22] that the binary alloy Mg–6Zn had the highest corrosion resistance (E_corr_(V) = 1.67, I_corr_(A) = 122, and corrosion rate (mm/year) of 2.78) compared to Mg–2Zn (E_corr_(V) = 1.86, I_corr_(A) = 210, and corrosion rate (mm/year) of 4.8) and Mg–10Zn (E_corr_(V) = −1.74, I_corr_ (µA) = 135 and corrosion rate (mm/year) of 3.08).

Paul et al. [23] examined novel Mg–Ca–Zn types with variable Ca and Zn percentages (Mg5Ca35Zn, Mg12Ca16Zn, and Mg15Ca22Zn). The hardness and Young’s modulus of the new alloys were 35% lower than those of the Mg5Ca35Zn alloy. In vitro degradation behavior in Hank’s balanced salt solution (HBSS) confirmed the existence of magnesium hydroxide (Mg(OH)_2_), hydroxyapatite (HAp), and ternary calcium magnesium zinc (Ca_2_Mg_6_Zn_3_) phases in the novel alloys compared to the previous Mg5Ca35Zn alloy. Zhang et al. [24] examined a Mg–0.2Ca–3Zn alloy with low content of alloying elements and different columnar structures in a NaCl degradation environment of 0.9% by weight. The corrosion test results indicated that the alloy with a cellular structure suffered homogenous corrosion and the lowest corrosion rate between 0.10 and 0.27 mm/y. Due to the protective corrosion product layer (CPF), the alloy with a cellular structure exhibited excellent corrosion resistance.

Zander et al. [25]. examined the microstructure of different Mg–Ca–Zn biodegradable alloys with varying Ca (0.6 to 1.6 wt.%) and Zn (wt.%) percentages (0.8 to 1.8 wt.%). In the Mg–0.6Ca system, alloying with 0.8% zinc caused a small reduction in grain size from 68 microns to 57 microns. Ternary alloys with Zn concentrations of more than 1.8% exhibited a grain microstructure with larger grains than alloys with Zn concentrations of 0.8%. Compared to the Mg–0.6 Ca alloys, the average grain size was increased by 21% for Mg–1.6Ca–xZn and by 32% for Mg–0.6Ca–xZn (x = 0.8, 1.8 wt.%).

**Figure 1:**
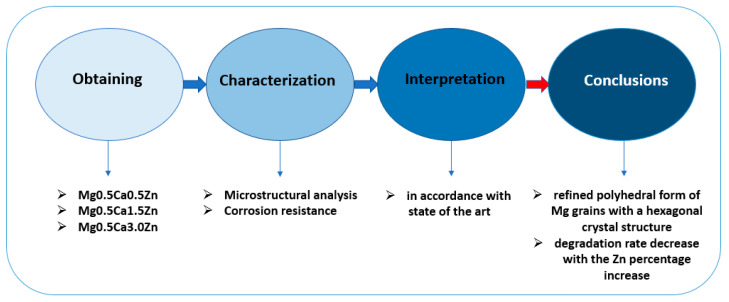
Schematic overview of the paper.

Due to the good degradation and biocompatibility aspects of zinc and calcium elements, these were chosen to obtain new alloys for medical applications. Elements such as magnesium, calcium, and zinc are essential for life, and do not affect the metabolism of the human body. Following the study of specialized literature and other studies carried out in other articles, three magnesium alloys were developed to contribute to improving the corrosion resistance and corrosion rate. Using a controlled atmosphere electric resistive melting furnace, three alloys were obtained: Mg0.5CaxZn (x: 0.5, 1.5, and 3.0 wt.%) and were investigated from the structural, electrochemical, and biodegradability points of view. This paper highlights the structure in correlation with the electrochemical response of a new experimental Mg-based biodegradable alloy. In Figure 1, the schematic overview of the paper is shown.

## 2. Materials and Methods

### 2.1. Obtaining Mg–Ca–Zn Biodegradable Alloys

Mg-98.5 wt.%, Mg–Zn (80 wt.%–20 wt.%), and Mg–Ca (85 wt.%–15 wt.%) were used as high-purity components and master alloys for the Mg–Ca–Zn alloying process [26,27]. To produce Mg–Ca–Zn alloys, a controlled environment electric resistive melting furnace (SY0002-2000W-1Kg) from the Faculty of Mechanics, Technical University Gheorghe Asachi, Iasi, Romania, was used. The raw materials were prepared for casting and dosed for each batch by weighing with an electronic balance. Table 1 shows the loads of the raw material used that resulted from the loading calculation for the experimental alloys. Ca (Mg–15%Ca), Zn (Mg–20%Zn), and 99.7% pure technical Mg master-alloys were used for ingot manufacturing. Cylindrical graphite crucibles, coated with refractory paint, were used with the following dimensions: outer diameter: 50 mm, inner diameter: 39 mm and height of 100 mm. The estimated material calculation to fill the crucibles was approximately 100 g.

Melting was performed by heating at 720 °C with holding for 5 min at this temperature for homogenization. During the melting operations, purging of the crucible with inert gas (Ar) was performed. Homogenization was achieved with a stainless-steel rod by constant mixing of the charge. The load calculations for the three experimentally programmed alloys are given in the table below.

### 2.2. Microstructural Analysis

In order to highlight the morphology of the experimental materials, the Mg–Ca–Zn biodegradable alloys were shown using two SEM microscopes. To make the samples more electrically conductive, a Luxor Au SEM COATER—CT-2201-0144 (Kusterdingen, Germany) was used to deposit a 5 nm coating of gold to the surface and cross-section of the samples. An SEM FEI Quanta 200 3D microscope (Brno, Czech Republic) with the following specifications was used: Low vacuum mode, LFD (large field detector), HV (high voltage): 20 kV, WD (working distance): 10 mm, comparable to previous research [28], and a Vega TESCAN LMHII SEM microscope (Brno, Czech Republic) with the following parameters [29]: secondary electrons (SE) detector, electron gun supply: 30 kV, high vacuum, and 15.5 mm working distance. X-ray diffractions were performed using a Panalytical Xpert PRO MPD 3060 (Almelo, The Netherlands) facility equipped with a Cu X-ray tube (K = 1.54051), 2Theta: 20°–90°, step size: 0.13°, time/step: 51 s, and a scan speed of 0.065651°/s in the reflection mode. Highscore Plus 3.0 was used to analyze the data and identify the phase components and their characteristics.

### 2.3. Corrosion Resistance

The laboratory alloys after mechanical grinding and ultrasound cleaning in technical alcohol for 30 min were immersed in simulated body fluid solution at 37 °C for five days. The samples were turned daily from side to side. During the immersion tests, using a micro pipette, the pH was corrected each 360 min with HCl 1 M to maintain the near 7.4 value. The experiments were realized in a simulated body fluid (SBF) solution made from (amount in 1000 mL): NaCl: 8.035 g, NaHCO_3_: 0.355 g, KCl: 0.225 g, K_2_HPO_4_–3H_2_O: 0.231 g, MgCl_2_–6H_2_O: 0.311 g, 1.0 M HCl: 39.0 mL, CaCl_2_ 0.292 g, Na_2_SO_4_ 0.072 g, ((HOCH_2_)3CNH_2_) 6.118 g, and 1.0 M HCl: appropriate amount for adjusting the pH at 7.4. The solution pH in contact with the experimental materials (first sample, respectively Mg0.5Ca0.5Zn with 2.82 cm^2^ exposed area; Mg0.5Ca1.5Zn: 3.08 cm^2^ and Mg0.5Ca3Zn: 2.42 cm^2^) was registered for 72 h (each minute) using Hanna HI98191 equipment. We followed the variation of pH in order to confirm the compounds and solution formation on the surface of the material. A Partner digital balance was used, after three measurements, to determine the samples’ mass before and after immersion. Being a three element system alloy and also given the possibility of contact with other metallic materials, the samples were subject to electro-corrosion. Electrochemical resistance of the experimental MgCaZn alloys was registered in SBF solution. A three electrode glass cell (work electrode WE, Pt electrode and saturated Calomel electrode) was used with a VoltaLab-21 potentiostat (Radiometer, Denmark) to analyze the linear and cyclic polarization curves in the SBF electrolyte. The samples’ response in terms of the potential and current were processed using the Volta Master 4 programs package. For the WE, the corrosion sample was isolated with Teflon so only a specific area was exposed to electrolyte, respectively 0.38 cm^2^. The electrolyte was continuously aerated with a magnetic stirrer in order to remove the gas bubbles that formed on the metal surface based on hydrogen elimination.

## 3. Results and Discussion

### 3.1. Microstructural Analysis

**Figure 2:**
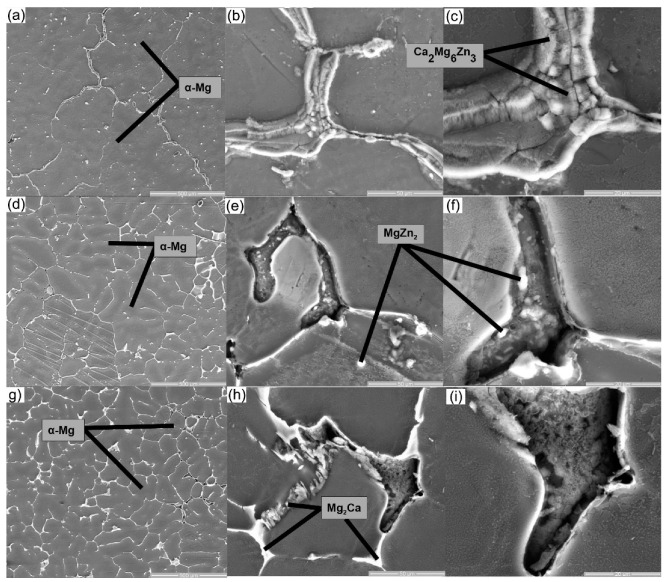
SEM images of the Mg–0.5Ca–xZn samples: (**a**–**c**): x = 0.5 wt.% Zn; (**d**–**f**): x= 1.5 wt.% Zn; (**g**–**i**): x= 3 wt.% Zn.

**Figure 3:**
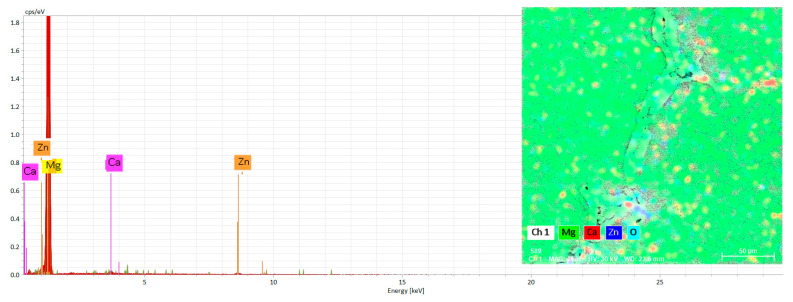
EDS spectra of the Mg–Ca–Zn alloys.

**Figure 4:**
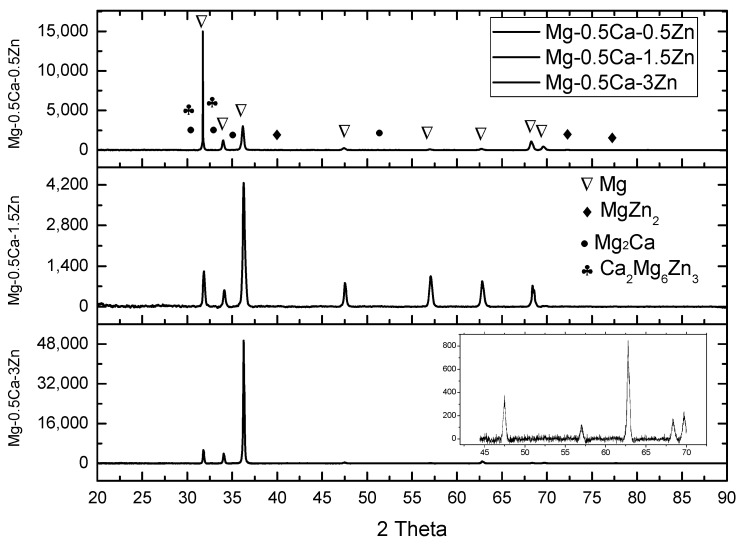
XRD patterns of the Mg–0.5Ca–xZn biodegradable alloys.

Scanning electron microscopy (SEM) studies were performed to validate the results of the XRD investigation. Figure 2 and Figure 3 show the SEM images and EDS element mappings of Mg–Ca–Zn alloys. In addition, the XRD data (see Figure 4), SEM micrographs (see Figure 2), and EDS results (see Table 2) revealed that the alloys were mostly constituted of a significant number of α-Mg phase and α-Mg + Mg_2_Ca + MgZn_2_ + Mg_6_Ca_2_Zn_3_ eutectic structures. Investigations of the microstructures suggest that the development of certain chemical compounds is associated with the formation of homogeneous microstructures. At the boundary between the magnesium grains, the Mg_2_Ca compounds formed a pellicular eutectic with magnesium. Progressive addition of zinc resulted in globular shapes with a tendency to separate and a distinct, rather uniform white color (see Figure 2). In addition, the SEM images indicated the presence of white dots representing Mg–Zn formations. It was discovered that increasing the Zn concentration led to microstructure refinement and a decrease in grain size. Figure 3 and Table 2 illustrate the surface areas of the specimens in five unique zones along with their relative means. The EDS-mapping (see Figure 3) of the Mg–Ca–Zn alloys demonstrates that the elemental distribution varies when Ca or Zn is added. Interdendritic interstices exhibited relatively high Zn contents, suggesting the precipitation of Zn-containing phases.

### 3.2. XRD Analysis

The results of the XRD analysis are shown in Figure 4. This pattern highlights the XRD spectra of the Mg–0.5Ca–xZn alloys. It can be seen that all of the alloys cast mainly had the following phases: α-Mg, Mg_2_Ca, and MgZn_2_. The increasing variation in the Zn concentration led to the formation of MgZn_2_. The main α-Mg (COD-pdf file: 96-901-3061) phase had a hexagonal structure and was identified with the following peaks: 32.42°, 34.59°, 36.58°, and 68.12°. Mg_2_Ca-type secondary phases (COD-pdf file: 96-431-3242) -(hexagonal structure) appeared at 59.51° and 64.15°, MgZn_2_-type secondary phases (COD-pdf file: 96-591-0079)-(hexagonal structure) were highlighted at angles of 28.85°, 30.97°, and 32.39°, respectively, and Ca_2_Mg_6_Zn_3_-type secondary phases, similar to [30], were observed at angles of 28.12° and 33.25°. The lattice parameters of the Mg–0.5Ca–xZn alloy phases are presented in Table 3. On account of a higher peak intensity at lower angles, the Mg peaks at the higher angles were not visible. The 40° ÷ 70° Theta diffractogram was inserted on the XRD graph at Mg–0.5Ca–3Zn to detail the specific compounds.

### 3.3. Electrochemical Analysis

The materials are proposed for medical applications in contact with different body fluids or parts. Mg from magnesium-based alloys reacts with water from the body fluid. The product formation is in conformity with the reaction (1):

Mg + 2H_2_O → Mg (OH)_2_ + H_2_ (1)

**Figure 5:**
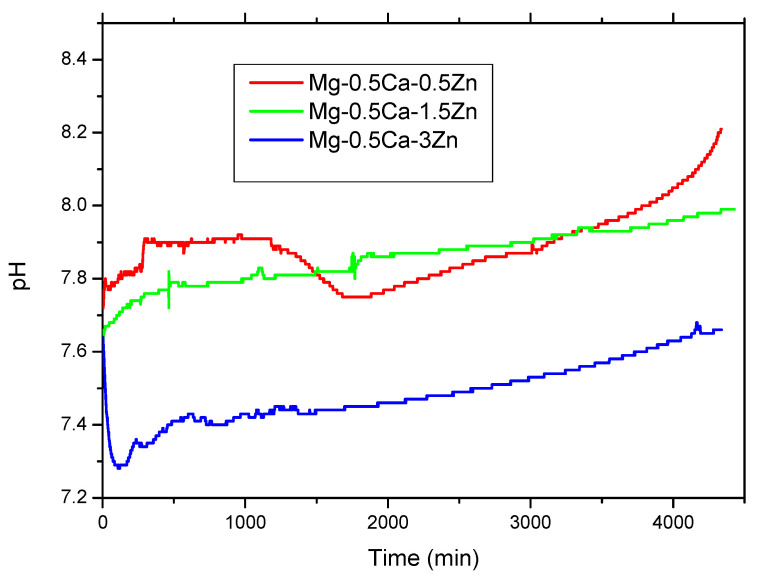
SBF solution pH variation after contact with the MgCaZn samples.

In electrolyte media possessing a high pH (>11.5), strongly basic solutions, Mg-hydroxide will provide protection against corrosion, behaving as a protective layer on the surface of Mg alloys, but at a pH < 11.5, it will promote the degradation of Mg-alloys in the physiological environment [31]. Since the pH at the implant–bone interface is 7.4 or lower, due to secondary acidosis resulting from metabolic processes and post-surgery resorption processes [32], the Mg (OH)_2_ layer will fail to provide effective protection against corrosion. Unfortunately, constant exposure to a high chlorine electrolyte in the physiological system will cause a fast degradation rate of the Mg implant surface in vivo. Figure 5 shows the pH variation in the SBF solution in contact with the experimental material (each of alloy was tested three times) at a 37 °C solution temperature. In all cases, at the beginning, an increase in the solution pH was observed based on reactions from Equations (1)–(5).

This behavior was observed in the first four hours after immersion and in continuation, a small reduction in the solution pH was related to acid formation during the local pitting corrosion of the surface. The pH of the solution in contact with Mg0.5Ca0.5Zn presented a strong variation between 1100 and 1700 h of immersion and the pH value can be attributed to the pitting corrosion of the surface after a few layers from the surface were removed. When increasing the percentage of Zn, we observed a stabilization of the solution pH with small variations and a general increase in the basic character of the solution and small reductions based on acid compound formation, a fact that confirms the controlled degradation process of Mg-based alloys.

The mechanism of the corrosion process of MgCaZn alloys with 0.5Ca and Zn with smaller percentages will mainly be based on the Mg corrosion behavior. The degradation process of Mg-alloys in contact with an environment (gaseous or liquid) is generally an electrochemical process, with a different behavior in solutions than in air [33,34]. The corrosion of Mg-alloys in the liquid solutions (like the biological environment, simulated body fluids, Ringer, etc.) is characterized by the reactions below [35]:

anodic reaction: Mg → Mg^2+^ + 2e^−^ (2)

cathodic reaction: 2H_2_O + 2e^−^ → H_2_ + 2OH^−^ (3)

2H_2_O + O_2_ + 4e^−^ → 4OH^−^ (4)

and compound formation: Mg^2+^ + 2OH^−^ → Mg(OH)_2_ (5)

The second reaction (2), which is of an anodic type, forms an important quantity of hydrogen and the cathodic reaction (4) accelerates the growth of the hydroxide protection layer. The passivation of the surface given by this layer is not sufficiently resistant, and through the subsequent polarization anodic reaction, will strongly participate in surface destruction [31]. This aspect is given to the corrosion of Mg-based alloys as a galvanic approximation. The layer of Mg(OH)_2_ growth on the surface of Mg-based alloys, in the biological environment, will lose its protective capacity under the influence of chlorine ions in the near area tissues. At the time the concentration of chlorine ions in the electrolyte solution exceeds 30 mmol/L, Mg(OH)_2_ will react to form a water-soluble MgCl_2_, thus accelerating the degradation process [36,37].

The surface behavior of the metallic material in contact with the aqueous solution is represented by the chemical reactions presented below:

Mg (OH)_2_ + 2Cl^−^ → MgCl_2_ (6)

Mg + 2H_2_O → Mg(OH)_2_ + H_2_ (7)

Mg + 2Cl^−^ → MgCl_2_ (8)

The growth of the MgCl_2_ layer on the metallic alloy surface will contribute to the reduction in the corrosion resistance, based on the average character of the solubility of the MgCl_2_ salt [38]. The main compounds that appeared after the interaction with the aqueous solution are biocompatible with the biological environment and without distinct cytotoxic effects [39]. The forming process of the compounds is complex and all the reactions occur in a matter of nanoseconds, and at the same time, the formation and evolution of hydroxyl ions increases the pH. Furthermore, calcium and phosphate ions will precipitate various calcium phosphates as a protective layer on the surface [18]. After contact with the SBF solution, the oxidation process of the alloy occurs with the formation of electrons, which will be consumed by cathodic reactions and the release of hydrogen gas and the hydroxide, with the formation of a protective layer on the surface [40].

**Figure 6:**
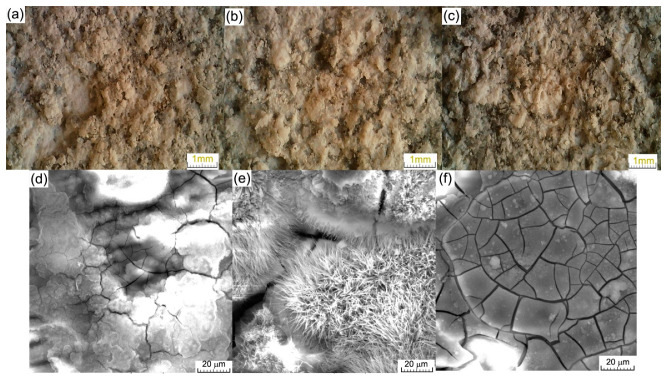
Surface statement after immersion in SBF at 37 °C with light microscopy images in (**a**–**c**) and electron scanning microscopy in (**d**–**f**).

Figure 6 shows the macroscale (a)–(c) and microscale (d)–(f) images of the surface after immersion. On the surface of the alloy, as seen in Figure 6a–c, a compact layer of different compounds was detected with variations in the thickness, cracks, and porosity. At the microscopic level, in Figure 6d–f, a continuous layer with micro-cracks and various shapes covered the entire surface, together with typical Mg-based oxides (Figure 6e). The degradation procedure will continue to be dependent on the passivation of the surface during the initial contact period, with the passivation layer rapidly dissolving and a new corrosion layer forming [41,42].

Using the formula from Equation (9), a corrosion rate based on mass loss was determined and given in Table 4, along with the mass variation in the samples after a 5 day immersion period. The zinc addition contributes to a reduction in the corrosion rate of the Mg-based alloy and decreased to a rate of 0.63 mm/year for Mg0.5Ca3Zn in static immersion conditions.

(9) $$CR=\frac{8.76\times 10^{4}W}{At\rho }$$

**Figure 7:**
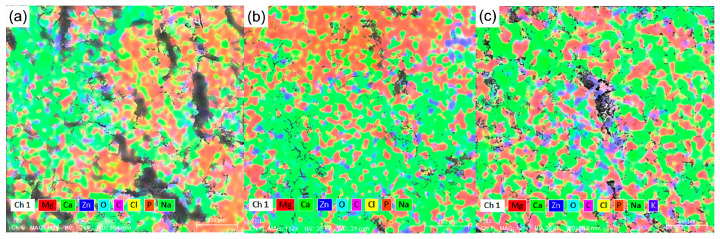
Elemental distribution on the surface after immersion in SBF: (**a**) Mg0.5Ca0.5Zn, (**b**) Mg0.5Ca1.5Zn, and (**c**) Mg0.5Ca3Zn.

Chemical elements were identified on the surface through X-ray energy dispersive spectroscopy (EDS) using automatic analysis mode after the alloy immersion in SBF solution at 37 °C for five days and after ultrasound cleaning in technical alcohol for 30 min. The element distributions are given in Figure 7a–c. The quantitative mass and atomic percentages of the elements identified on the surface are compared in Table 5. For all samples in Figure 6, the surface was mainly covered by corrosion products and only small areas of Mg could be observed, especially on sample Mg0.5Ca3Zn. The distribution of the oxides, chlorides, and different salts, carbonates, and phosphates were observed in all cases. The analysis of the surface was performed after ultrasonic cleaning of the alloys, which means that the metallic surface and all of the compounds are interconnected and bound, and the detachment of these will be performed with a few layers, depending on how deep the surface is penetrated, of the MgCaZn alloy as degradation products.

The surface of the materials was mainly oxidized and the presence of C, Cl, Na, and P was also identified. Calcium was present in a small percentage as well as zinc, their signals being masked by the oxides, salts, and carbonates layer.

Usually, Zn is used as an addition element for Mg, being itself a solution for biodegradable metallic materials and has the ability to remove H ions from solution. When Zn is used as in Mg–Zn, the following reactions would take place at the same time with reactions (1)–(7), as already above-mentioned:

Zn (s) + 2 H_2_O (aq) → Mg (OH)_2_ (s) + H_2_ (g) (10)

Oxidation Reaction: Zn(s) → Zn_2_ + (aq) + 2e^−^ (11)

In the case of a SBF solution that contains groups like HCO^3−^ and HPO_4_ ^2−^, the corrosion compounds are also based on Mg/Ca carbonates and phosphates that might increase the precipitations on the surface of Mg, confirmed by the chemical analysis (Table 5 and Table 6) of the surface as well as the compounds that passed in the electrolyte solution. The presence of these compounds has the effect of decreasing the degradation rate of Mg-based alloys. The main reactions are:

Mg^2+^ (or Ca^2+^) + OH^−^ + HCO_3_^−^ + (n − 1) H_2_O → Mg (or Ca) CO_3_·nH_2_O (12)

3Mg^2+^ (or Ca^2+^) + 2OH^−^ + 2HPO_4_^2−^ + (n − 2) H_2_O → Mg_3_ (or Ca) (PO_4_)_2_·nH_2_O (13)

The spread of the corrosion compounds of Mg is mainly general during the degradation process. The formation and growth of compounds such as Ca_3_(PO_4_)_2_ appear in any part of the surface and preferentially around the Ca-based compounds from the alloy. A different compound that can appear on the surface such as Mg_3_(PO_4_)_2_ may grow homogeneously around the pitting hole-like areas. The principal motive is that a big percentage of Mg ions avoids the formation of Ca_3_(PO_4_)_2_ [43,44]; it is, as a consequence, easier for Mg_3_(PO_4_)_2_ to precipitate on the alloy surface. During the degradation process after the isolation of the alloy surface with a protective coating of Mg_3_(PO_4_)_2_, the growth nucleation of Ca_3_(PO_4_)_2_ takes place and a non-uniform distribution of Ca_3_(PO_4_)_2_ forms at the product layer [18]. The repetitive process finishes each time by completing the degradation of the Mg-based alloy through the equilibrium between the growth and formation and dissolution of degradation products, aside from the conversion of the active layer into a passive one [41].

**Figure 8:**
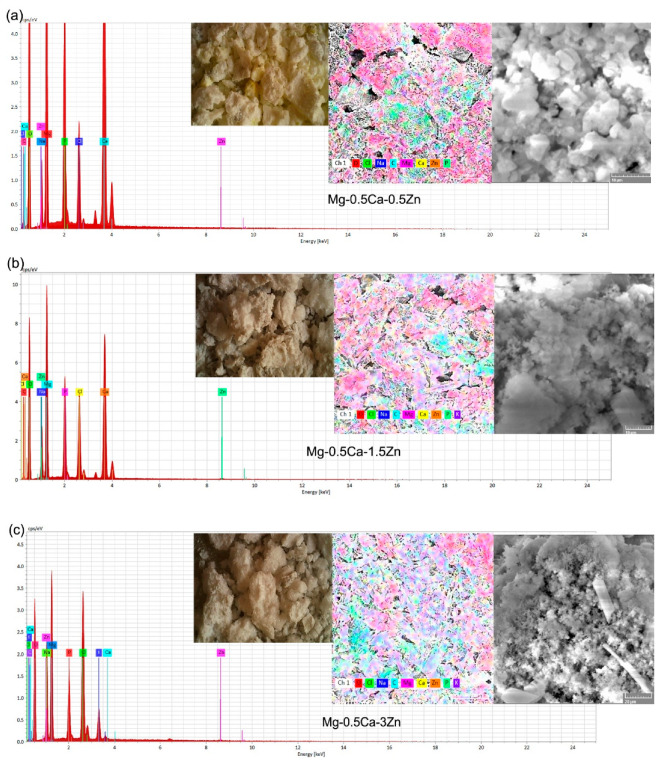
Morphology and chemical element distribution of the compounds that passed in the SBF solution after a five day immersion period: (**a**) Mg0.5Ca0.5Zn, (**b**) Mg0.5Ca1.5Zn, and (**c**) Mg0.5Ca3Zn.

From the immersion solution, we separated the compounds from the alloy–SBF interaction using filter paper and performed the optical, electronic analysis of the morphology and EDS detection for the elemental analysis. The general aspect of the compounds is given in Figure 8a–c through light microscopy. At the macroscale, the compounds were compact with millimeter dimensions with different colors (Figure 8). The conglomerates observed at the macroscale formed from small dimensional corrosion parts. At the microscale (Figure 8a–c), we observed compounds or agglomerations of compounds with a minimum radius of 740 nm, maximum of 3.35 μm, and an average of 1.69 μm with a St. Dev of 0.6 for the compounds extracted from the solution with the alloy Mg0.5Ca0.5Zn. For the alloy with 1.5 Zn, the minimum radius of the compounds was around 500 nm, the maximum was 2.24, with an average of 1.35 μm with a standard deviation of 0.3. For the last investigated chemical composition, respectively, Mg0.5Ca3Zn, the smallest conglomerate was around 200 nm, the maximum was 1.2 μm, and had an average of 520 nm with a 200 nm standard deviation. The addition of Zn actively participated in the decrease in the dimensions of the corrosion compounds, a fact that is helpful for reducing the risk of internal blockage, injuries, etc.

The chemical composition of the compounds was determined using an EDS detector and the mass and atomic percentages are given in Table 6. In the case of the first alloy, no compounds with potassium were present in the solution and as seen in Table 5, no K X-ray energy was registered on the surface. The percentage of phosphorus from the corrosion compounds also increased with the Zn percentage from the alloy, so alloying with Zn favors a phosphorus reaction with the metallic material.

The addition of zinc in biomedical Mg-based implants presents the advantage of being absorbable by biological bodies after dissolution from the alloy material due to corrosion. High quantities of Zn have the potential to be corrosive in nature if ingested [36]. When zinc (Zn^+2^) ions react with hydrochloric acid (HCl), ZnCl products are formed, which have been shown to damage parietal cells lining the inside stomach walls.

Compared to the mass loss/gravimetric evolution technique, the potentio-dynamic technique provides the possibility to evaluate the thermo-dynamic tendency or spontaneity of the corrosion reaction (E_corr_), corrosion kinetics (I_corr_), and ultimately, to calculate the corrosion rate V_corr_. based on Faraday’s law. All samples were ultrasonically cleaned and dried before the test.

Table 7 presents the main parameters of the electro-corrosion resistance tests of MgCaZn alloys in the SBF solution. The tendency of improving the corrosion resistance observed from the immersion test was confirmed for electro-corrosion resistance. The electro-corrosion rate was similar for all of the materials with a slightly smaller value of the alloy with 3% Zn, which can be attributed to the zinc oxide passivation effect on the corrosion resistance of the material. Reactions presented activities at the cathode and anode branches with appropriate values (β_c_ and β_a_).

Adding a Ca element to magnesium can improve the material general pitting resistance and decrease the self-corrosion current density of the Mg-alloy. However, a small percentage of Ca will attenuate the micro battery effect of the Mg alloy and the electro-corrosion resistance is improved [45].

Alloying with Zn will definitely influence the electro-corrosion resistance of Mg-based materials. Based on the phase diagram, the maximum limit of solid solubility of Zn in Mg is, in general obtaining conditions, around 6 wt.%. It is usually added with other chemical elements as reinforcement parts of the Mg matrix, increasing the strength of the material. The cathodic hydrogen evolution reaction of the Mg alloy would be inhibited when its content of Zn is less than 3 wt.%. At the same time, adding more than 5 wt.% Zn seems to negatively affect the corrosion resistance of the material [45].

**Figure 9:**
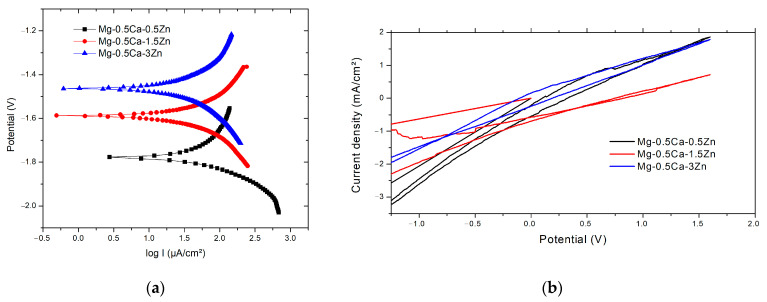
Electro-corrosion resistance test (**a**) Tafel representation and (**b**) cyclic curves.

Continuing the addition of Zn would accelerate the anodic dissolution of Mg. Anodic and cathodic reactions occur with similar intensities and provoke, with same the importance, the degradation of the material (Table 7 and Figure 9). The corrosion potential was bigger for the alloy with a smaller Zn percentage, respectively, Mg0.5Ca0.5Zn.

**Figure 10:**
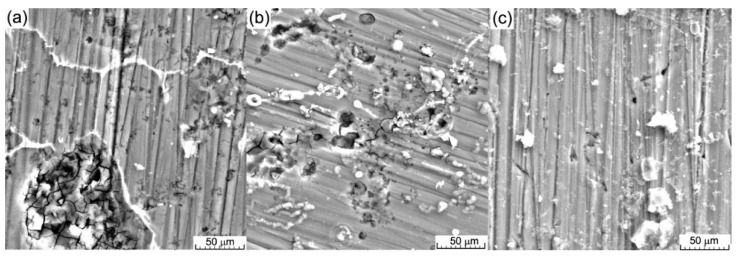
SEM image of the surface after the electro-corrosion test in SBF solution: (**a**) MgCa0.5Zn, (**b**) MgCa1.5Zn, and (**c**) MgCa3Zn.

All cyclic diagrams (Figure 9b) were similar and presented a generalized corrosion type with almost no differences between the branches. For the biodegradable materials, the pitting type corrosion started the corrosion of the surface (Figure 10) in an accelerated rhythm, and the pitting sites very quickly connected and the corrosion gained a generalized aspect, as observed and confirmed by the immersion tests.

The SEM images of the surface of the alloys after the electro-corrosion test and ultrasonic cleaning presented a corroded surface with pitting sites (Figure 6a), which were more, bigger, and denser on samples with 0.5 and 1.5 wt.% Zn. On all surfaces, corrosion products could be observed. As can be observed from Figure 6b, the pitting holes tended to connect and form a big crack in the material, usually under the corrosion product layer, which will enhance the degradation of the alloy surface.

In this electrolytic alloy system, Mg is less noble than Zn and Ca, which conducts a matrix dissolution, mainly releasing Mg, but also Ca and Zn (alloying elements in solid solution in the matrix) into the metal/aqueous medium interface. The release of these metal ions at the interface will eventually change the surface potential to positive values and the anodic polarization will increase. The Zn oxide that forms on the surface will improve the corrosion resistance of the surface.

## 4. Conclusions

The experimental biodegradable Mg-based alloys Mg–0.5Ca–0.5Zn, Mg–0.5Ca–1.5%Zn, and Mg–0.5Ca–3Zn were investigated in this study. In conclusion, the authors identified the importance of Zn in terms of the microstructure and corrosion resistance. Due to the addition of Zn, the scanning electron microscopy and XRD patterns revealed a refined polyhedral form of Mg grains with a hexagonal crystal structure. Due to the addition of Ca, the Zn particles (MgZn_2_-white compounds) appeared to have a globular morphology, and at the Mg grain boundary, the Mg_2_Ca eutectic compound was generated.

From the point of view of the corrosion and electro-corrosion of the MgCaZn alloy, the zinc alloying element to the MgCa biodegradable alloy influenced the corrosion properties, improving the corrosion resistance in the SBF solution. The reactivity of the Mg0.5CaZn alloy was smoothened, with the Zn percentage increase confirmed by the pH variation in the SBF solution, which presented smaller variations with the Zn percentage increase in the alloy (0.5Zn compared with 3 wt.% Zn). Additionally, the degradation rate decreased as the Zn percentage increased (up to 3 wt.% Zn), and the corrosion products were conglomerates of nano-compounds with different dimensions and shapes based mainly on oxides, carbonates, chlorides, salts, and phosphates.

The electro-corrosion behavior was similar for all three alloys tested, presenting a general corrosion on the surface, which initially started with pitting-like sites that grew very fast, connected to each other, and finished with the detachment of the surface layer and the passing of surface compounds to the electrolyte solution. The Tafel diagram variations present a smaller corrosion potential for the alloy with more Zn wt.%, anodic, and cathodic reactions with similar intensities, and likewise, a smaller corrosion rate for the Mg0.5Ca3Zn alloy compared with Mg0.5Ca0.5Zn.

In order to determine the precise cell viability and behavior of laboratory animals, the investigation will continue with in vitro and in vivo testing of these alloys.

## Figures and Tables

**Table 1:** Calculation of the metallic load for the experimental alloys Mg–0.5Ca–xZn.

Alloy	Mg [g]	Mg–15Ca [g]	Mg–20Zn [g]
Mg–0.5Ca–0.5Zn	94.17	3.33	2.5
Mg–0.5Ca–1.5Zn	89.17	3.33	7.5
Mg–0.5Ca–3Zn	81.67	3.33	15

**Table 2:** Chemical composition results by the Bruker EDS method.

Alloy	Mg [wt.%]	Ca [wt.%]	Zn [wt.%]
Mg–0.5Ca–0.5Zn	99.05	0.43	0.52
Mg–0.5Ca–1.5Zn	98.10	0.51	1.39
Mg–0.5Ca–3Zn	96.22	0.63	3.15

Note: EDS analysis performed in five separate areas.

**Table 3:** Lattice parameters of the Mg–0.5Ca–xZn alloy phases.

Compound	Space Group	Crystal System	a (Å)	b (Å)	c (Å)	α (°)	β (°)	γ (°)	Cell Volume (10^6^ µm^3^)	RIR
Mg	P63/mmc	Hexagonal	3.2420	3.2420	5.2660	90	90	120	47.93	4.10
Mg_2_Ca	P63/mmc	Hexagonal	5.8350	5.8350	18.8970	90	90	120	557.19	2.37
MgZn_2_	P63/mmc	Hexagonal	5.1500	5.1500	8.4800	90	90	120	194.78	3.67
Ca_2_Mg_6_Zn_3_	P63/mmc	Hexagonal	9.7250	9.7250	10.148	90	90	120	-	-

**Table 4:** Corrosion rate determined by each mass loss of samples subjected to immersion tests and subsequent cleaning in an ultrasonic bath (Immersion time for 5 days).

Immersion Time/State of the Samples	Mg0.5Ca0.5Zn	Mg0.5Ca1.5Zn	Mg0.5Ca3Zn
Initial mass (mg)	399.9	533.4	449.5
Mass after immersion (mg)	405.1 (+5.2)	529.6 (−3.8)	450.8 (+1.3)
Mass after ultrasonic cleaning (mg)	385.3 (−14.6)	527.3 (−6.1)	447 (−2.5)
CR (mm/year)	3.62	1.30	0.63

Standard Deviation: ±0.1 mg.

**Table 5:** Chemical composition of the surface after immersion in SBF and ultrasound cleaning (30 min in technical alcohol, the results represent an average value of three determinations on a 1 mm^2^ surface).

Elements/ Sample	O [%]		Mg [%]		C [%]		Cl [%]		Na [%]		P [%]		Ca [%]		Zn [%]	
	wt.	at.	wt.	at.	wt.	at.	wt.	at.	wt.	at.	wt.	at.	wt.	at.	wt.	at.
Mg0.5Ca0.5Zn	58.55	64.39	25.91	18.76	8.9	13.03	3.56	1.77	1.86	1.42	0.86	0.49	0.22	0.1	0.14	0.04
Mg0.5Ca1.5Zn	58.64	64.11	25.09	18.06	9.45	13.77	2.75	1.36	2.74	2.09	0.76	0.43	0.09	0.04	0.1	0.05
Mg0.5Ca3.0Zn	47.55	56.61	14.53	11.39	8.38	13.28	12.16	6.53	9.12	7.56	5.01	3.08	0.09	0.04	0.22	0.06
Detector Error %	3.8		1.7		2.2		0.14		0.25		0.07		0.05		0.04	

Note: On sample Mg0.5Ca3Zn, a percentage of 2.94 wt.% of K was identified; St. dev: O: ±2.3; Mg: ±1.2; C: ± 1.1; Cl: ±0.25; Na: ±0.2; P: ±0.2; Ca: ±0.05; Zn: ±0.05.

**Table 6:** Chemical composition of the compounds passed in the electrolyte solution.

Chemical Elements/Alloy	Cl %		O %		Na %		C %		Mg %		P %		Ca %		Zn %		K %	
	wt.	at.	wt.	at.	wt.	at.	wt.	at.	wt.	at.	wt.	at	wt.	at.	wt.	at.	wt.	at.
Mg–0.5Ca–0.5Zn	34.46	20.22	32.52	42.29	15.52	14.05	9.81	16.99	6.96	5.95	0.68	0.45	0.03	0.02	0.02	0.01	-	-
Mg–0.5Ca–1.5Zn	10.60	5.67	48.47	57.49	7.81	6.45	8.06	12.73	16.21	12.66	5.98	3.66	0.16	0.07	0.21	0.01	2.51	1.22
Mg–0.5Ca–3.0Zn	6.91	3.74	48.63	58.34	7.47	6.23	6.99	11.18	15.31	12.09	9.75	6.04	0.02	0.01	0.28	0.08	4.63	2.27
EDS det. error %	1.34		5.0		1.21		3.01		0.48		0.06		0.08		0.01		0.12	

St Dev.: Cl: ±1.0; O: ±2.5; Na: ±0.3; C: ±0.25; Mg: ±0.75; P: ±0.1; Ca: ±0.02; Zn: ±0.01; K: ±0.12.

**Table 7:** Electro-corrosion parameters (average values from three determinations).

Alloy/Electro-Corrosion Parameters	E_corr_ (I = 0) [mV]	I_corr_ [µA/cm]	Rp [kohm cm^2^]	V_corr_ [mm/Y]	βc [mV/dec]	βa [mV/dec]
Mg–0.5Ca–0.5Zn	−1781	49.37	0.885	7.96	−156	418
Mg–0.5Ca–1.5Zn	−1589	41.83	1.23	8.11	−380	404
Mg–0.5Ca–3.0Zn	−1458	32.26	1.47	7.03	−276	287
